# Motion corrected silent ZTE neuroimaging

**DOI:** 10.1002/mrm.29201

**Published:** 2022-04-05

**Authors:** Emil Ljungberg, Tobias C. Wood, Ana Beatriz Solana, Steven C. R. Williams, Gareth J. Barker, Florian Wiesinger

**Affiliations:** ^1^ Department of Neuroimaging Institute of Psychiatry, Psychology & Neuroscience, King's College London London UK; ^2^ Department of Medical Radiation Physics Lund University Lund Sweden; ^3^ GE Healthcare Munich Germany

**Keywords:** motion correction, neuroimaging, RUFIS, silent MRI, ZTE

## Abstract

**Purpose:**

To develop self‐navigated motion correction for 3D silent zero echo time (ZTE) based neuroimaging and characterize its performance for different types of head motion.

**Methods:**

The proposed method termed MERLIN (Motion Estimation & Retrospective correction Leveraging Interleaved Navigators) achieves self‐navigation by using interleaved 3D phyllotaxis k‐space sampling. Low resolution navigator images are reconstructed continuously throughout the ZTE acquisition using a sliding window and co‐registered in image space relative to a fixed reference position. Rigid body motion corrections are then applied retrospectively to the k‐space trajectory and raw data and reconstructed into a final, high‐resolution ZTE image.

**Results:**

MERLIN demonstrated successful and consistent motion correction for magnetization prepared ZTE images for a range of different instructed motion paradigms. The acoustic noise response of the self‐navigated phyllotaxis trajectory was found to be only slightly above ambient noise levels (<4 dBA).

**Conclusion:**

Silent ZTE imaging combined with MERLIN addresses two major challenges intrinsic to MRI (i.e., subject motion and acoustic noise) in a synergistic and integrated manner without increase in scan time and thereby forms a versatile and powerful framework for clinical and research MR neuroimaging applications.

## 1 INTRODUCTION

MRI has developed into an indispensable tool for neuroscience research and constitutes a fundamental step for many clinical pathways in neurology. Remaining obstacles to further adoption and clinical use include its high cost, the loud and narrow‐bore environment, and long scan times resulting in pronounced motion sensitivity.^1^ A more patient‐friendly and quiet scanning environment where the MR imaging is tolerant to patient motion will further boost its utility and reduce the amount of aborted and repeated scans.

The acoustic noise produced by the MRI scanner can reach levels up to 130 dB,^2^ making it a necessity to wear hearing protection, and can be a major source of discomfort and distress especially for vulnerable patient populations.^3^, ^4^ Motion artifacts on the other hand are problematic in all medical imaging techniques, but especially in MRI using high resolution and long scan times. From a clinical perspective, motion artifacts can obscure pathology, requiring re‐acquisition of the scans and thus imposing additional costs to the hospital and extra burden for the patients. Studies have estimated the added cost to hospitals, due to failures in patient motion management, to be on the order of $100,000/scanner/year.^5^, ^6^ In research studies, motion artifacts can affect morphological and functional measurements,^7^, ^8^ which is problematic since movement inside the scanner is known to be higher for some groups, for example, pediatric, geriatric, and neurodegenerative patients.^9^, ^10^ Addressing the issues of acoustic noise and patient motion offers a multitude of advantages including reduced costs (to health‐care providers) due to repeated scans, improved clinical decision‐making, higher quality neuroscience research, improved compliance and overall satisfaction for both clinical patients and research participants. Many solutions have been proposed to tackle acoustic noise and motion artifacts, separately, but not in the form of an integrated solution as described in this work.

The acoustic noise produced by the MRI scanner originates from Lorentz forces in the gradient coils and the surrounding shielding material.^11^ To reduce the acoustic noise, the rate of gradient switching has to be reduced, typically by smoothing the gradient waveforms.^12^ An alternative approach is to use zero echo time (ZTE) imaging,^13^, ^14^ for which RF excitation is performed during the readout gradient allowing rapid acquisition of the free induction decay (FID) along a center‐out radial spoke in 3D k‐space. Following data acquisition, the gradient direction is updated directly to that needed for the next spoke (i.e., without ramping down and back up again in between). If the spokes are arranged along a smooth path with only small directional changes in between neighboring spokes, ZTE imaging can be performed with minimal gradient switching in a virtually silent manner.^15^

In 3D radial ZTE imaging, all readouts originate in the center of k‐space, resulting in a sampling density which decreases toward the edge of the k‐space support. The repeated acquisition of the center of k‐space reduces motion sensitivity via averaging, although it does not eliminate it entirely.^16^ For datasets acquired with full Nyquist encoding at the edge of the k‐space support, multiple images can be reconstructed from independent data at a smaller radius. Radial imaging therefore naturally lends itself to self‐navigation, which has been demonstrated in numerous studies.^17^, ^18^, ^19^, ^20^, ^21^, ^22^ A general advantage of self‐navigation is that it is independent of the contrast mechanism and does not add additional elements to the sequence, different from dedicated navigators that are inherently noisy. To enable self‐navigation, spokes must be arranged such that navigator images can be reconstructed at regular time intervals, which in 2D can be achieved using a golden angle increment between spokes.^23^ This can also be extended to 3D applications using multi‐dimensional golden means,^22^, ^24^, ^25^ however this approach does not ensure that spokes are arranged to minimize the gradient switching, and hence acoustic noise. Another approach for 3D radial self‐navigation is the spiral phyllotaxis,^26^, ^27^ which can be used to create a set of smooth spirals, called interleaves, each defining the directions of a series of radial spokes, such that when combined still provides approximately uniform sampling density.

In this work, we combine 3D ZTE imaging with self‐navigated motion correction based on spiral phyllotaxis k‐space sampling for silent and motion corrected neuroimaging. First, we describe the implementation of the 3D phyllotaxis trajectory to create navigator images and characterize the resulting image quality and acoustic noise profile via phantom experiments. Second, we present a framework for retrospective rigid body motion estimation using a sliding window reconstruction. The presented sampling and motion correction method is termed MERLIN for Motion Estimation and Retrospective correction Leveraging Interleaved Navigators. Finally, we present examples of in vivo motion correction using MERLIN with a variety of instructed motion paradigms in a group of healthy volunteers. The code used for motion estimation and correction, and image analysis is available on GitHub, along with simulated example data.

## 2 METHODS

All MR experiments were conducted on a 3T GE MR750 scanner (GE Healthcare, Waukesha, WI) using a 3D radial ZTE pulse sequence.^13^ Phantom experiments used the Alzheimer's Disease Neuroimaging Initiative (ADNI) MRI phantom,^28^ in combination with a 12‐channel receive head coil (GE Healthcare, Waukesha, WI), while in vivo experiments were carried out using a 32‐channel receive brain coil (Nova Medical, Wilmington, MA). The body coil was used for RF excitation in all cases. The transmit‐receive switching time was assumed to be 30–40 μs, resulting in a central spherical k‐space gap (also referred to as the deadtime gap^29^) of two to three samples radius for the considered imaging bandwidth (BW) of ±31.25 kHz. 3D non‐Cartesian image reconstruction was performed using the RIESLING toolbox,^30^ including gridding and iterative SENSE reconstruction. The study received ethical approval as part of an overarching technique development project (HR‐20/21‐21138), and all participants provided written consent prior to participation.

### 2.1 Part 1: self‐navigated ZTE

#### 2.1.1 An interleaved 3D phyllotaxis trajectory

ZTE sequences typically use straight spokes; therefore, we define the ZTE k‐space trajectory as the path traced by the 3D center‐out spoke endpoints. To reconstruct an image with isotropic matrix size MAT from a 3D radial acquisition, $N_{t}=\pi \cdot \mathrm{MA}T^{2}\;$ evenly distributed spokes are required to satisfy the Nyquist sampling criterion at the edge of k‐space. Arranging those spokes in a pseudo‐random manner permits self‐navigation via reconstruction of low‐resolution navigator images from smaller continuous subsets of the acquired data.^31^ To maintain silent operation, the gradient step between subsequent spokes must be small. The natural combination of these two requirements is a spoke distribution composed of multiple sparse spirals, called interleaves. The number of spokes per interleave determines the spatiotemporal encoding of the navigators.

**FIGURE 1:**
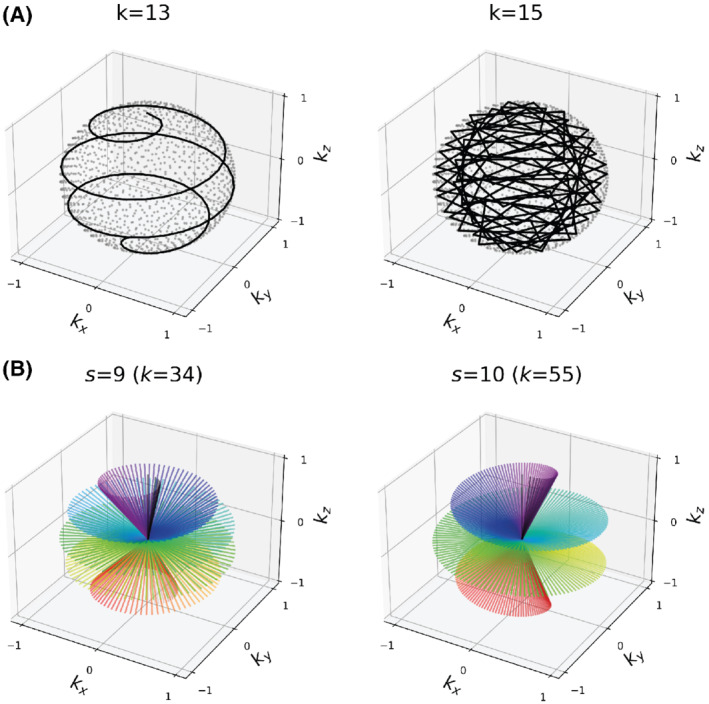
A, Illustration of two trajectories (each 1300 spokes) using different subsampling values *k*. A smooth phyllotaxis spiral interleave is only obtained with *k* being a Fibonacci number (e.g., *k*=13). B, Illustration of two interleaves (each 384 spokes) using different smoothing factors (*s*). A higher value of *s* produces smaller azimuthal increments (i.e., smoother and quieter trajectory) but larger polar increments (i.e., higher undersampling and streaking)

Several methods exist to produce interleaved spiral trajectories.^27^, ^32^, ^33^ We adopted the 3D spiral phyllotaxis method because of its pseudo‐random sampling and elegant mathematical description.^27^ In the original phyllotaxis trajectory design, the full set of spokes is first obtained via constant increments of (i) the azimuthal angle by the golden angle ($\phi_{G}\approx 137.5^{\circ }$) and (ii) the *z*‐coordinate such that the first and last spoke align along the north and south pole of the k‐space sphere. The full set of spokes is then divided into interleaves by choosing every *k* ^th^ spoke with *k* being a Fibonacci number as illustrated in Figure 1A. It can be shown, that for a subsampling factor *k*, where *k* is the *s* ^th^ Fibonacci number, the azimuthal increment is given by

(1) $$\Delta \phi_{k}=\frac{2\pi }{g^{s}}$$

with g≅1.618 being the golden ratio^23^, ^34^ (cf. Supporting Information section SI.1, which is available online). For example, the eighth Fibonacci number is 21, which gives $\Delta \phi_{8}=7.7^{\circ }$. We will use the term “smoothness factor” for the value of *s*. As can be seen from Figure 1B, higher *s* will give smaller azimuthal increments (i.e., quieter acquisition), but larger polar angle (θ) increments (i.e., higher undersampling and streaking). The trade‐off between interleaf sparsity and navigator image quality will be investigated in this section.

The full ZTE trajectory is produced by sequentially stacking the spiral phyllotaxis interleaves following the subsampling scheme described above. The direction of spoke i in interleaf j in spherical coordinates is, hence, given by

(2) $$\begin{array}{cc} & \phi_{i,j}=\left(i\cdot k+j\right)\cdot \phi_{G},\;i=0\ldots N_{s}-1\\ & z_{i,j}=1-\left(i\cdot N_{i}+j\right)\cdot \Delta z,\Delta z=\frac{2}{N_{s}\cdot N_{i}-1},\\ & \;j=0\ldots N_{i}-1\\ & \theta_{i,j}=\text{acos}z_{i,j} \end{array}$$

where $N_{s}$ is the number of spokes per interleaf, $N_{i}$ is the number of interleaves, and $N_{t}=N_{i}\cdot N_{s}$ is the total number of spokes. Subsequent interleaves are rotated by $\phi_{G}$ and will, thus, fill the largest remaining gap in k‐space. When $N_{i}=k$ near perfect uniformity is achieved. Note that we use a different modulation of the polar angle compared to Piccini et al., to produce an isotropic field of view^35^, ^36^ (cf. Supporting Information Figure S2).

#### 2.1.2 Sequence integration

The interleaved 3D spiral phyllotaxis trajectory was implemented into a 3D radial ZTE sequence including a short

**FIGURE 2:**
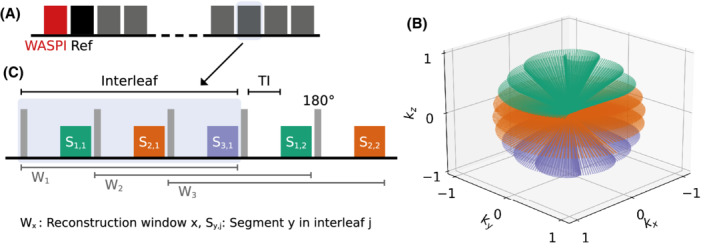
A, Schematic of the segmented ZTE pulse sequence starting with the WASPI acquisition followed by multiple interleaves, the first one serving as the reference for the registration. B, Example of a single phyllotaxis spiral interleaf consisting of three segments indicated by different colors. C, Example of a segmented acquisition where each segment is preceded by a 180° inversion pulse and a T_1_ recovery period (TI) to produce T_1_ contrast. Any set of three adjacent segments can be reconstructed to produce a navigator image, indicated by the reconstruction window W_x_

WASPI acquisition^37^ to support filling the deadtime gap in navigator and final images. The WASPI data were acquired before all other data, that is, in temporal proximity to the reference position (cf. Figure 2A), and used a gradient scaling factor of 0.125, resulting in only a marginal extension of the overall scan time. The ZTE acquisition is implemented in a segmented manner with optional magnetization preparation (e.g., inversion recovery, arterial spin labeling, T_2_ preparation, magnetization transfer, diffusion, etc.). Each segment acquires a certain number of spokes per segment which can be flexibly adjusted dependent on the contrast preparation and ZTE scan parameters used. Between segments, the gradients are slowly ramped down and back up again to the direction of the first spoke in the following segment, thus ensuring silent operation even with larger changes in spoke direction between interleaves. Following the segmented structure of the ZTE pulse sequence, each phyllotaxis spiral interleave is divided into an integer number of segments (e.g., three segments per phyllotaxis spiral interleave in Figure 2B).

**FIGURE 3:**
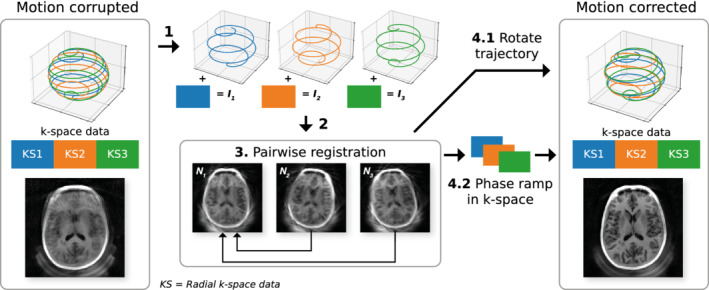
Flowchart of the self‐navigated MERLIN motion estimation and retrospective correction framework. For illustration, the full trajectory consists of only three interleaves. The trajectory and k‐space data are split into separate interleaves (1; *I_1_, I_2_, I_3_*) and reconstructed into navigator images (2; *N_1_, N_2_, N_3_*), which are then registered to a reference state to estimate rigid body motion (3). Rotational motion is corrected for by rotating the trajectory (4.1) and translational motion is corrected for by applying a linear phase ramp to the k‐space data (4.2). The corrected k‐space trajectory and data are then combined and reconstructed into a single motion corrected image

The self‐navigated MERLIN motion correction framework is illustrated in Figure 3. The interleaves are reconstructed into separate low‐resolution navigator images which are pairwise registered to a reference, here the first navigator image. Corrections, described in Section 2.2, are applied to the k‐space trajectory and raw data, which are then combined to produce a single, high‐resolution, motion corrected image.

#### 2.1.3 Navigator image reconstruction

Acquiring extra WASPI segments for each phyllotaxis spiral interleave to fill the deadtime gap would significantly increase the temporal footprint for each navigator and overall scan duration. Instead, we first created sensitivity maps free of dead‐time gap artifacts by reconstructing only the WASPI data and dividing each channel image by the root sum‐of‐square combination.^38^ We then used these maps in an iterative conjugate gradient SENSE (cgSENSE)^39^, ^40^ reconstruction for each navigator image. This implicitly fills the deadtime gap, in the same way cgSENSE fills undersampled regions at the edges of k‐space,^41^ without including low spatial frequencies from the WASPI data which may be mis‐matched due motion. Tikhonov regularization was used to avoid noise‐amplification and reduce streaking in the background.^42^ The Pipe sample density correction method^43^ and Töplitz embedding^44^ was used to accelerate the reconstruction process.

#### 2.1.4 Data acquisition

A phantom experiment was carried out to characterize the influence of trajectory parameters on image quality and acoustic noise. Data were acquired with smoothing factors s={9,10,11} and $N_{s}=\left\{2\ldots 8\right\}\cdot 256=\left\{512,\ldots ,2048\right\}$ spokes per phyllotaxis spiral interleave, at a readout of BW = ±31.25 kHz, TR = 1.8 ms, prescribed resolution 1 × 1 × 1mm^3^, and FOV = 192 × 192 × 192 mm^3^. RF excitation was performed using a 12 μs hard pulse with flip angle (FA) = 3°. A single navigator was then reconstructed from each acquisition at a resolution of 3 × 3 × 3 mm^3^.

The WASPI acquisition, used to obtain sensitivity maps, consisted of 2048 uniformly distributed spokes, that is, not phyllotaxis. The acoustic noise was measured using an MR compatible microphone placed on the scanner bed approximately 50 cm distant from the head coil. Measurements were taken over 30 s and the A‐weighted equivalent sound levels (LAeq) were recorded. To study the influence of the readout BW on the acoustic noise, additional scans were acquired with BW = ±15.6 and ±25 kHz, using *s=10*.

A reference ZTE scan was acquired using a continuous trajectory with $N_{t}=37\;376$ spokes (effective undersampling of 3.1), but otherwise identical parameters to the ±31.25  kHz BW acquisition and reconstructed using the same process as the navigator images. Image quality of the individual navigator images was assessed and compared to the reference using the mean structural similarity index measure (mSSIM) within a spherical mask covering the phantom.^45^

### 2.2 Part 2: temporal stability of motion estimates

The quality of the motion corrected image is limited by the accuracy and precision of the motion parameters obtained from the registration algorithm which in turn depend on the object features and their appearance in the navigator images.^46^ For this reason, the temporal stability was assessed via in vivo experiments instead of phantom experiments, which would miss relevant anatomical features.

#### 2.2.1 Sliding window reconstruction of IR‐prepared ZTE

Different to the phantom scans in Part 1 which used native ZTE, all in‐vivo experiments were acquired using an inversion recovery (IR) prepared ZTE sequence to achieve T_1_ contrast with an inversion time of TI = 450 ms,^47^ followed by a readout of 384 spokes per segment. Multiple segments ($N_{\mathrm{seg}}$) are thus required to obtain enough spokes for accurate navigator reconstruction, as discussed in Part 1. Figure 2C illustrates an IR‐ZTE sequence with three segments per phyllotaxis spiral interleaf. Navigator images can be reconstructed from any subset of consecutive spokes with sufficient 3D k‐space coverage, either from individual interleaves or by mixing subsequent interleaves in a sliding window manner, also known as view sharing. Sliding window reconstruction has been used in multiple studies with non‐Cartesian imaging to improve the temporal resolution.^23^, ^48^, ^49^ A natural choice is to step the window forward by the extent of a full segment resulting in a total of $N_{\mathrm{nav}}=\left(N_{i}-1\right)\cdot N_{\mathrm{seg}}+1$ potential navigator images.

#### 2.2.2 Motion estimation and correction

The navigator images were registered to the first navigator using a 3D rigid body transformation which applies a centered rotation ($R\left(\alpha_{x},\alpha_{y},\alpha_{z}\right)$) followed by a translation ($\bar{\Delta }=\left(\Delta_{x},\Delta_{y},\Delta_{z}\right)$).^50^ The 3D non‐selective ZTE imaging further eliminates out‐of‐slice, or out‐of‐slab imperfections known from retrospective motion correction in 2D, or 3D slab‐selective imaging.

Retrospective 3D rigid body motion correction (i.e., translation and rotation) can be performed either in image space or k‐space. Here, we apply all correction factors to k‐space data to avoid image interpolation artifacts.^17^, ^19^, ^51^

A rotation around the center of the image corresponds to the same rotation of the k‐space coordinates; hence, the corrected trajectory is given by

(3) $${\bar{k}}_{\text{corr}}=R\left(\alpha_{x},\alpha_{y},\alpha_{z}\right)\cdot \bar{k}$$

where $\bar{k}\;$ are the k‐space coordinates. According to the Fourier shift theorem, translations in image space are equivalent to a linear phase ramp in k‐space and can thus be applied to each k‐space datapoint as

(4) $$y_{\text{corr}}=y\cdot \exp\left(i2\pi \cdot \bar{\Delta }\cdot {\bar{k}}_{\text{corr}}\right)$$

with y being the radial k‐space data.

For a sliding window reconstruction, a particular segment may contribute to multiple navigators, each of which will result in a different set of registration parameters. We chose to assign the registration parameters derived from each window to its middle segment. That way the motion parameters represent the average position within the navigator window and will, thus, correct for position but not higher‐order velocity, or acceleration effects. After correcting each segment separately, the data are combined into a single dataset for reconstruction of the final high‐resolution image.

#### 2.2.3 Stability tests

An in vivo dataset was acquired from a single healthy volunteer using the IR‐ZTE sequence with $N_{\mathrm{seg}}=\left\{2,3,4\right\}$ for a total of 768, 1152 and 1536 spokes per interleaf. Navigator duration was 2.3, 3.5, and 4.7 s, respectively. The acquisition parameters were: FOV = 192 × 192 × 192 mm^3^, resolution = 1 × 1 × 1 mm^3^, TI = 450 ms, 12 μs rectangular RF excitation pulse with FA = 3°, BW = ±31.25 kHz, TR = 1.8 ms, and $N_{t}\approx 93\;312$; resulting in a slight undersampling of 1.25 (relative to Nyquist) and total scan time of ∼5 min. Navigator images were reconstructed at 3 mm isotropic resolution and motion correction was performed as described in the previous section. The WASPI data, acquired before the reference navigator (Figure 2A), consisted of 1536 spokes. The choice of navigator resolution was based on previous work using ZTE for T_2_‐prepared fMRI, which used 1024 spokes per volume, reconstructed at 3 mm.^52^ Reconstructing navigators at a lower resolution would reduce the computational burden but potentially degrade motion correction quality. A comparison of motion correction quality as a function of navigator resolution is provided in Supporting Information section SI.2.

Variations in the motion estimates were assessed by calculating the mean and SD of the magnitude of the translation and rotation vectors, and a frequency analysis to identify periodic oscillations. Since some drifts in position are inevitable, despite instructions to lie still, we applied detrending by convolving with a uniform window, with a length of 51 segments, before calculating the SD along the time series.

ZTE sequences can detect signals from the head coil and padding around the head,^53^ which will remain static when the subject moves. Therefore, we performed automatic brain extraction on the first navigator image^54^ and evaluated the cost function for the registration only within the brain mask, see Supporting Information section SI.3.

### 2.3 Part 3: in vivo validation with different motion paradigms

In the final experiment, multiple volunteers were scanned with the IR‐ZTE protocol and sliding window image reconstruction with $N_{s}=1152$ spokes per interleaf, based on results from Part 2. All other acquisition parameters were the same as in the previous experiment (Section 2.2.3), duration of each navigator was 3.5 s, and duration of the WASPI acquisition was 4.7 s. For each participant, two static scans were acquired in the beginning of each session, followed by four instructed motion paradigms, including small and big rotations in the axial plane, nodding motion in the sagittal plane (similar to swallowing), and continuous side‐to‐side motion for 1 min.

The full datasets were reconstructed before and after motion correction using a self‐calibrated iterative SENSE approach^55^ with total generalized variation (TGV) regularization ($\lambda =2\cdot 10^{-5},N_{\mathrm{it}}=8)$.^56^ The regularized iterative reconstruction reduces noise and background streaking arising from the incoherent angular undersampling of the motion‐corrected trajectory. Since the WASPI data were acquired at the very start of the sequence, the head was assumed to be in the same position as the reference navigator, which immediately followed this and, thus, could be used to fill the deadtime gap explicitly.

Image quality before and after motion correction was assessed by calculating the average edge strength (AES)^57^ and the mSSIM. The AES measures the image sharpness in a single image; a reduction in AES is expected with motion. The mSSIM compares two images to indicate how similar they are, mSSIM = 1 corresponds to perfect agreement. With a static image as the reference, we expect an increase in the mSSIM after motion correction for images with motion. First, all scans for each subject were registered to the first static scan (the reference), using the same registration framework as described for the motion correction. For each scan, the mSSIM and the percentage change in AES were calculated relative to the reference before and after motion correction. Both the AES and mSSIM were calculated within a brain mask extracted from the reference.^54^ For further details about AES and mSSIM, we refer the reader to Supporting Information section SI.4. For visual assessment, RF bias correction was applied using estimated coil sensitivity information.^58^

## 3 RESULTS

### 3.1 Results Part 1: trajectory and navigators

**FIGURE 4:**
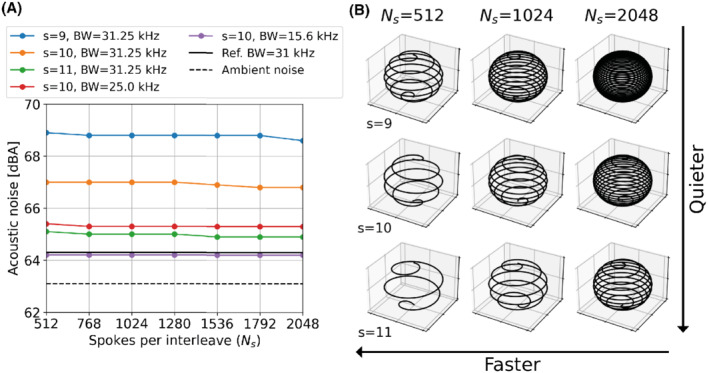
A, Acoustic noise measurements (LAeq) for different smoothing factors, and readout BWs as a function of the number of spokes per interleave (*N*_s_), with comparison to a non‐interleaved reference acquisition and the ambient noise levels. B, Example of trajectories with different number of spokes per interleave and smoothing factors as used in (A)

Figure 4A shows how the acoustic noise decreased with increasing *s*, as expected given the correspondingly smaller angular steps between spokes (Equation 1). Despite reducing the number of spokes per interleaf for a given *s,* resulting in a sparser trajectory with larger steps in *z* (see Figure 4B), the acoustic noise only changed by ≤0.3  dBA, demonstrating that azimuthal gradient switching is the dominant factor. Reducing the readout BW, which results in reduced gradient strength and increased TR, decreased the acoustic noise; with $N_{s}=1024$, and *s*=10 , the increases above ambient were 3.9, 2.2, and 1.1 dBA, respectively for BW = ±31.25, ±25.0, ±15.6 kHz.

**FIGURE 5:**
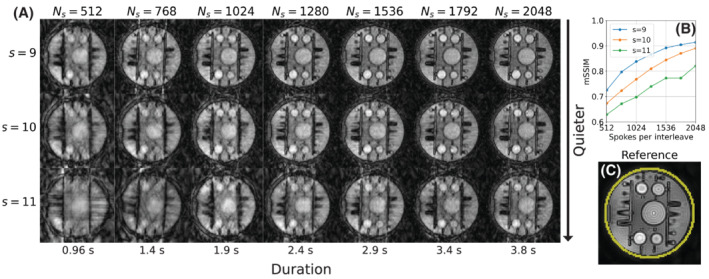
A, Comparison of image quality in the phantom data for different number of spokes and trajectory smoothing factors. B, Quantitative assessment of image quality using mSSIM in the navigator images relative to the reference in (C), with the SSIM calculated within the mask outlined in yellow

The navigator image quality was affected both by the number of spokes and the smoothing factor, as shown in Figure 5. The mSSIM was higher for lower smoothing factors, which yield more uniform but acoustically louder trajectories. For subsequent experiments, we chose *s*=10 as a trade‐off between image quality and acoustic noise.

### 3.2 Results Part 2: temporal stability

**FIGURE 6:**
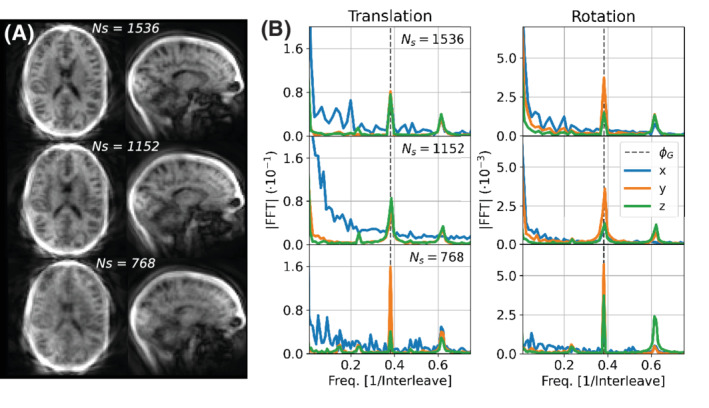
A, Example of navigator images with different number of spokes per interleave. B, Frequency analysis of the estimated motion parameters from a static acquisition with detrending, showing a clear peak at the frequency corresponding to the golden angle $\phi_{G}$

Representative navigator images for each acquisition are shown in Figure 6, and the corresponding motion traces can be found in Supporting Information Figure S5. The variations in the motion estimates were highest with $N_{s}=768$ ($\left|\bar{\Delta }\right|=0.18\pm 0.07\;\mathrm{mm},\left|\bar{\alpha }\right|=0.32\pm 0.10^{\circ }$), but similar for $N_{s}=1152\;\left(\left|\bar{\Delta }\right|=0.13\pm 0.05\;\mathrm{mm},\;\left|\bar{\alpha }\right|=0.21\pm 0.06^{\circ }\right)$ and $N_{s}=1536\;\left(\left|\bar{\Delta }\right|=0.14\pm 0.05\;\mathrm{mm}\;\left|\bar{\alpha }\right|=0.22\pm 0.07^{\circ }\right)$. The frequency analysis of the motion trace (Figure 6B) shows a clear peak at the golden angle ($\phi_{G}$) frequency. Based on these results, we chose $N_{s}=1152$ for all subsequent in vivo experiments as a good compromise between motion estimate quality and temporal resolution.

### 3.3 Results Part 3: in vivo validation

**FIGURE 7:**
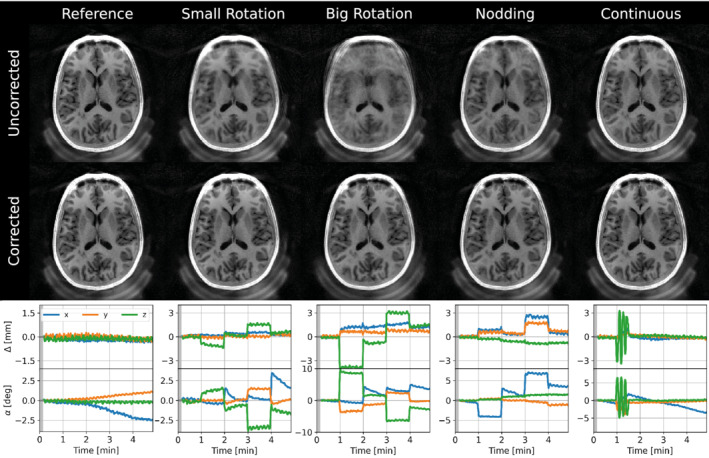
Axial slices and motion estimates from subject 3 demonstrating clearly improved image quality after motion correction. The gray region in the time series indicates the short time window at the beginning of the acquisition which is not motion corrected including dummy segments and the low resolution WASPI acquisition. The padding from the head rest is visible posterior to the head; a unique feature of ZTE acquisitions which have increased sensitivity to materials with ultra‐short T_2_. Images have been bias field corrected and windowed for optimal viewing quality

**FIGURE 8:**
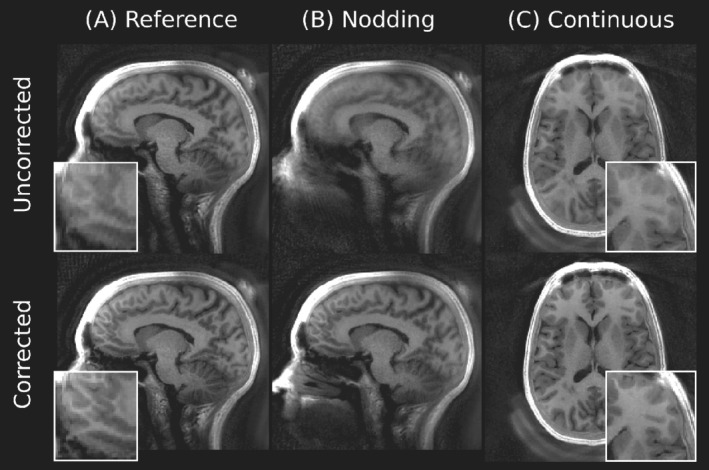
Additional views from subject 3 before and after motion correction. A, The reference image, without intended motion, shows improvement after motion correction due to unintentional x and y rotations (see Figure 7). B, Improvements for nodding motion are best seen in the sagittal plane. C, Improvements for continuous motion are best appreciated in the zoomed frontal lobe region

**FIGURE 9:**
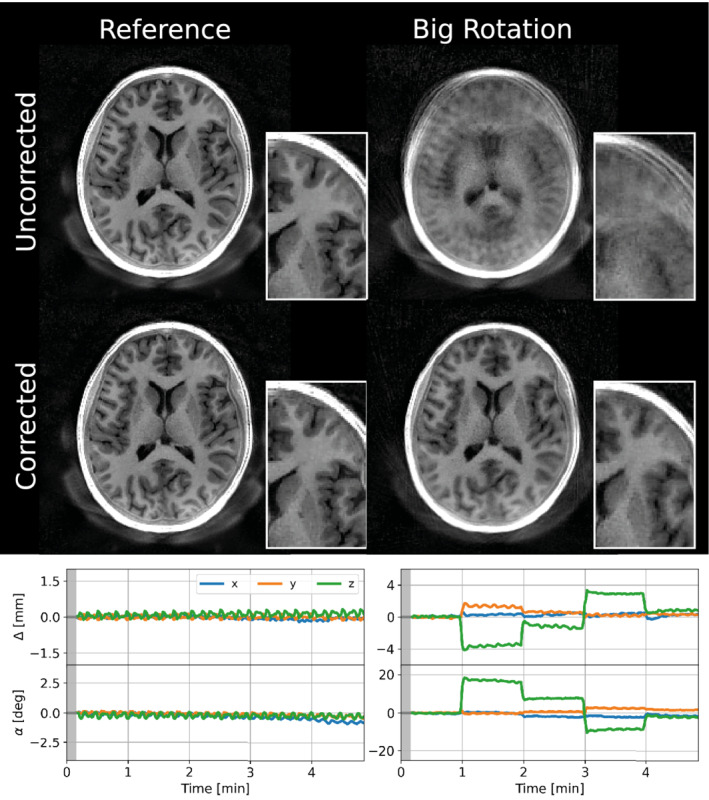
Axial slices and motion estimates from subject 1 demonstrating the improvement in image quality after motion correction, with rotations up to 20°. The gray region in the time series indicate the short time window of the acquisition which is not motion corrected including dummy segments and the low resolution WASPI acquisition

Representative examples of the estimated motion parameters and corrected images for all the instructed motion paradigms in one subject are shown in Figure 7, demonstrating that MERLIN can (i) successfully extract the motion traces and (ii) reconstruct images in a self‐navigated manner with greatly improved quality. Figure 8A shows improved image quality in the reference image where the subject unintentionally rotated their head slightly. Figure 8B shows the improvement from nodding in the sagittal plane, and Figure 8C the more subtle improvement in the axial plane from continuous motion. Figure 9 shows that axial rotations up to 20° can be successfully corrected. Additional axial and sagittal slices from all subjects are presented in Supporting Information Figures S6‐S8. Videos showing the motion navigator time series from subject 3 (Supporting Information Videos S1‐S3) illustrate how head motion is resolved during data acquisition but do also demonstrate flickering streaking artifacts between frames.

**FIGURE 10:**
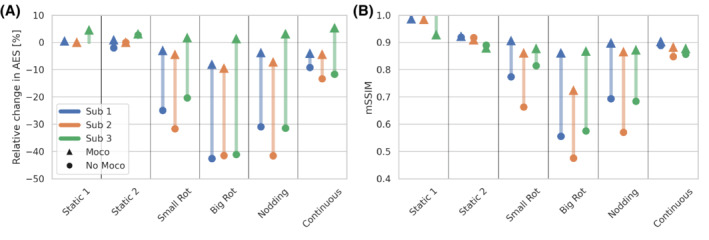
Quantitative assessments of the improvement in image quality after motion correction measured using the AES (A) and the mSSIM (B) both evaluated relative to the first, non‐motion corrected, static scan

Quantitative assessment of image quality in Figure 10 shows similar trends for all subjects, with improvements in the AES for both static and instructed motion acquisitions. The mSSIM increased after motion correction, approaching the mSSIM of the second static acquisition, indicating a near perfect correction.

## 4 DISCUSSION

In this work, we have presented a method for self‐navigated, retrospective motion correction, called MERLIN, which is based on silent 3D ZTE imaging combined with phyllotaxis k‐space sampling. The technical feasibility of MERLIN has been demonstrated for native, FLASH‐type ZTE in phantom experiments and T_1_ magnetization prepared ZTE in healthy volunteers for different instructed head motion paradigms. Similar, although loud, self‐navigated, motion corrected 3D radial imaging methods have been described by Kecskemeti et al. in their MPnRAGE method,^20^ and Lee et al., for UTE imaging.^22^

### 4.1 Trajectory characteristics

In Part 1, we evaluated the utility of 3D phyllotaxis sampling for ZTE to produce low resolution navigator images continuously throughout the acquisition. As expected, the image quality increased with higher number of spokes per interleaf, as this increases both the SNR and the sampling density in k‐space. A higher trajectory smoothing factor produces a smoother trajectory with fewer revolutions, that is, larger polar increments, which resulted in decreased image quality (Figure 5). However, the smoother trajectory has lower gradient switching and is, therefore, quieter; *s* = 9 was ∼4 dBA louder than *s* = 11. Given a fixed smoothing factor, the azimuthhal angle step is constant, and only the step size in *z* changes with the total number of spokes, which was shown to have minimal effect on the acoustic noise (Figure 4). We, therefore, concluded that *s* = 10 was a reasonable compromise between image quality and acoustic noise, and that ∼1000 spokes would be required for accurate navigator reconstruction.

### 4.2 Temporal stability in vivo

In the second experiment, we performed in vivo scans with 768, 1152, and 1536 spokes per interleave (with *s* = 10 fixed) to evaluate temporal stability. We observed the highest variability in translation and rotation estimates with 768 spokes. Frequency analysis of the rotation and translation estimates revealed a clear peak at a frequency equivalent to the rotation by the golden angle $\phi_{G}.$ This is unlikely to represent genuine motion; instead, we hypothesize that since subsequent interleaves are rotated by the golden angle, any streaking pattern (inevitably present in highly undersampled radially acquired data) and slab profile effects will rotate accordingly and may thus be responsible. This hypothesis is further supported by Supporting Information Videos S1‐S3, showing flickering between frames which we believe is caused by the continuously rotating phyllotaxis sampling pattern.

Oscillations with a known frequency in the motion estimates could be corrected by filtering, albeit at the risk of filtering out genuine movements with the same frequency. However, the amplitude of the observed oscillation is small (<0.4 mm and <0.4°), and we did not observe any degradation in image quality after motion correction for the in vivo results shown in Part 3. Future work will investigate alternative image reconstruction methods to reduce variability between navigators, but also the possibility of using a Kalman filter, as suggested in previous studies, for reducing this oscillation as well as noise in the position estimates.^59^, ^60^

### 4.3 Sliding window motion correction in vivo

In the third and final experiment, we demonstrated the in vivo efficacy of MERLIN in a small group of healthy volunteers with different instructed motion paradigms. While the exact magnitude of motion was not controlled, we observed similar reduction in image quality (AES and mSSIM) for all subjects. The degradation was largest for the big rotation and least for the short burst of continuous movement. In all cases, the image quality was markedly improved after motion correction, both visually (Figures 7, 8, 9) and quantitatively (Figure 10). For the third subject, we observed a decrease in the mSSIM after motion correction for the first static scan. This does not indicate reduced image quality, rather the opposite, given that the AES increased and, thus, the mSSIM is reduced since the image is sharper after motion correction (Figure 8). The mSSIM for the second static scan was similar for all subjects, before and after motion correction, with values around 0.9. We do not expect a perfect match between these images given random noise in the images; thus, we would not expect an mSSIM larger than ∼0.9 for the motion corrected cases either; it essentially serves as a baseline for the best‐case scenario.

MERLIN is limited to relatively slow, rigid body, motion. Here, we demonstrate good motion correction for piece‐wise constant motion, as expected from changing position due to discomfort, swallowing or coughing. Motion during a single navigator (3.5 s) will produce blurring and reduce registration quality, but with sliding window reconstruction, we can better identify when step‐like changes in position occur. Residual within‐navigator blurring could be addressed using a real‐time rejection scheme, where navigators with blurring above a certain threshold are reacquired.^60^

The efficacy of the motion correction is fundamentally limited by the accuracy and precision of the rigid body motion estimation. In our in vivo stability measurements, we found the variability of the translation motion estimates extracted from the 3 mm low‐resolution navigator images to be $\left|\bar{\Delta }\right|=0.13\pm 0.05\;\mathrm{mm}$, which is well below the intended image resolution of the IR‐ZTE acquisitions, that is, 1 mm. While the performance of the rigid body motion estimation is primarily dependent on the MR acquisition parameters of the navigator images, that is, resolution and SNR, it also relies on characteristics of the imaged object and the registration algorithm used and hence is difficult to assess in general terms.^46^, ^61^

The increase sensitivity to materials with short T_2_s, such as the head pads and the plastic coil housing (Figure 7), is problematic for motion correction since they remain static in the scanner frame of reference while the head is moving, thus contradicting the assumption of rigid body motion. We therefore masked out the head using automated brain extraction, which relies on good navigator image quality. This could be an issue in applications with low brain‐to‐skull contrast, such as proton density weighting.^62^ A more robust solution would be using pads with no detectable MR signal, such as inflatable air pads.

### 4.4 Advantages and limitations of MERLIN with ZTE

The main benefit of ZTE highlighted in this work is the near silent operation from the slow gradient switching, which also reduce eddy currents to a minimum. An additional benefit is minimal phase accumulation during the rapid data acquisition. At ultra‐high field strengths, head motion can alter the susceptibility‐induced *B*_*0*_ field inhomogeneities, and thus produce k‐space data with inconsistent phase and T_2_* decay when acquired with a non‐zero echo time.^63^ Given a sufficiently short readout and TE = 0, this problem is largely avoided.^64^

The MERLIN motion correction framework was demonstrated for T_1_ magnetization prepared ZTE but can be extended to other ZTE‐based pulse sequences including other types of magnetization preparation such as T_2_,^52^, ^65^ magnetization transfer,^66^ MRA,^67^ diffusion,^68^ and parameter mapping.^69^ With native FLASH‐type ZTE, further applications include variable FA T_1_ mapping,^70^ and PD‐weighted ZTE for bone imaging^62^ and synthetic CT conversion for PET/MR attenuation correction and radiation therapy planning.^71^ Similarly, MERLIN could be extended to Looping Star^72^ for quiet functional BOLD imaging,^73^, ^74^ and anatomical T_2_^*^ and susceptibility weighted imaging. MERLIN could also find applications in other parts of the body where the unique aspect of short‐T_2_ imaging could be used, such as bone or lung imaging.^64^, ^75^ Motion outside the brain is in many cases non‐rigid, which could be addressed by masking out an area where rigid body motion is expected,^76^ or more generally using local autofocusing for non‐rigid body motion correction.^77^

The auto‐calibrated coil sensitivity mapping is essential in MERLIN, here extracted from an initial WASPI acquisition, but could be substituted for other methods such as PETRA and HYFI.^78^, ^79^ The sensitivity maps were then used to implicitly fill the deadtime gap for each navigator during an iterative reconstruction process. This method has no time penalty but is limited to relatively small deadtime gaps,^41^ that is, fast transmit‐receive switching or low gradient amplitudes. Algebraic reconstruction can also be used without a time penalty, but it requires opposing spokes which was not supported by our trajectory. Higher BWs will require alternative strategies for filling the deadtime gap, or k‐space based registration methods.^17^, ^80^

A single set of sensitivity maps will not be strictly valid at different head positions. After motion correction, k‐space will be composed of data acquired with different sensitivity profiles, resulting in a final image with an average effective sensitivity profile. Accurate image reconstruction, and implicit deadtime gap filling, will therefore rely on moderate motion. Other methods, such as aligned SENSE which address this issue, could be investigated in future studies for motion corrected ZTE.^81^, ^82^

Finally, ZTE is inherently a non‐selective 3D technique, which has advantages and limitations. The main advantage with regard to motion correction is that we can correct for motion in 3D including out‐of‐slice motion, which is a common problem in 2D methods. However, non‐selective excitation requires encoding of a large FOV to avoid aliasing artifacts, thus potentially increasing scan time. This disadvantage can partly be reduced using oversampling along the spoke and parallel imaging with localized receive coils. The limitations of ZTE are most pronounced in body imaging, where a higher readout BW, thus larger deadtime gap, is required to avoid chemical shift artifacts.^64^ Body imaging also benefits from anisotropic FOV,^83^ which is not possible with the present implementation of MERLIN.

## 5 CONCLUSIONS

MERLIN is a method for self‐navigated, retrospective motion correction using 3D radial phyllotaxis k‐space sampling. It has the unique advantage of near silent operation when combined with a 3D ZTE pulse sequence, thus providing a solution for two major problems in MRI: subject motion and acoustic noise. The method was successfully applied for T_1_ weighted inversion‐recovery ZTE imaging in a group of healthy volunteers performing a range of instructed motion paradigms, with only a benign increase in acoustic noise (<4 dBA above ambient). MERLIN can be combined with any magnetization prepared ZTE sequence as it only requires modification to the sampling trajectory and does not impact any other sequence elements. Ultimately, we expect that MERLIN will guide the way toward silent and motion corrected neuroimaging examinations, including both structural and functional imaging.

The analysis code used for motion estimation and correction are provided on Github https://github.com/emilljungberg/pyMERLIN (#6074a6d). Code for reproducing Figures 1, 4, SI.1, and SI.2 are available in a separate Github repository, together with an example of how to run MERLIN with a simulated 3D Shepp‐Logan phantom https://github.com/emilljungberg/merlin_mrm (#9c17409). The data presented in this study may be accessed by contacting the authors directly.

## Supporting information


**Video S1.** (*SI_Video_S3_Bigrot.mp4*) Video of reconstructed navigator images and motion estimates from subject 3 performing a big rotation.

**Video S2.** (*SI_Video_S3_Nodding.mp4*) Video of reconstructed navigator images and motion estimates from subject 3 performing a nodding motion.

**Video S3.** (*SI_Video_S3_Continuous.mp4*) Video of reconstructed navigator images and motion estimates from subject 3 performing a fast continuous motion.

**Figure S1.** Azimuthal angle increment as a function of spoke subsampling factor. When *k* is a Fibonacci number, the angular increment ($\Delta \phi_{k})$ is small, which is required to maintain silent acquisition
**Figure S2.** Voronoi diagram which measures the sampling density in k‐space. **A** and **B** compares the 3D spiral phyllotaxis trajectory as formulated by Piccini et al. **(A)** with square root modulation of the polar angle θ, resulting in non‐uniform sampling density, and the trajectory used in MERLIN **(B)** with a cos^−1^ modulation, resulting in a linear z‐gradient and uniform sampling density for isotropic field of view.
**Figure S3.** Investigation of motion correction quality as a function of navigator resolution. **(A)** Motion corrected images from the big rotation paradigm, showing reduced motion correction quality with lower navigator resolution. **(B)** Quantitative analysis of image quality using the Average Edge Strength relative to the 3 mm experiment for each motion paradigm. A clear trend to reduced edge strength, i.e., increased blurring, is observed for lower navigator resolution.
**Figure S4.** Example of brain mask used for registration. The mask, here shown by outline only, is twice dilated to also cover the skull but to still exclude the signal from the headrest (white arrows) which will confound the registration since it does not move with the head, resulting in a non‐rigid motion.
**Figure S5.** Expanded version of main Figure 6, investigating motion correction stability. **(A)** De‐trended motion traces and representative navigator images from acquisition with different number of spokes per interleaf. **(B)** FFT analysis of the detrended motion traces showing a peak at the golden angle frequency.
**Figure S6.** Representative axial and sagittal slices from subject 1, from all motion paradigms, together with the estimated motion traces. The gray region in the time series indicates the short time window at the beginning of the acquisition which is not motion corrected including dummy segments and the low resolution WASPI acquisition.
**Figure S7.** Representative axial and sagittal slices from subject 2, from all motion paradigms, together with the estimated motion traces. The gray region in the time series indicates the short time window at the beginning of the acquisition which is not motion corrected including dummy segments and the low resolution WASPI acquisition.
**Figure S8.** Representative axial and sagittal slices from subject 3, from all motion paradigms, together with the estimated motion traces. The gray region in the time series indicates the short time window at the beginning of the acquisition which is not motion corrected including dummy segments and the low resolution WASPI acquisition.
