# Supplementary material for: Motion corrected silent ZTE neuroimaging
Source: Magn Reson Med. 2022 Apr 5;88(1):195–210. doi: 10.1002/mrm.29201 (PMC9321117; doi:10.1002/mrm.29201)
Supplement: Supplementary file 4 — Figure S1. Azimuthal angle increment as a function of spoke subsampling factor. When k is a Fibonacci number, the angular increment (Δϕk) is small, which is required to maintain silent acquisition Figure S2. Voronoi diagram which measures the sampling density in k‐space. A and B compares the 3D spiral phyllotaxis trajectory as formulated by Piccini et al. (A) with square root modulation of the polar angle θ, resulting in non‐uniform sampling density, and the trajectory used in MERLIN (B) with a cos−1 modulation, resulting in a linear z‐gradient and uniform sampling density for isotropic field of view. Figure S3. Investigation of motion correction quality as a function of navigator resolution. (A) Motion corrected images from the big rotation paradigm, showing reduced motion correction quality with lower navigator resolution. (B) Quantitative analysis of image quality using the Average Edge Strength relative to the 3 mm experiment for each motion paradigm. A clear trend to reduced edge strength, i.e., increased blurring, is observed for lower navigator resolution. Figure S4. Example of brain mask used for registration. The mask, here shown by outline only, is twice dilated to also cover the skull but to still exclude the signal from the headrest (white arrows) which will confound the registration since it does not move with the head, resulting in a non‐rigid motion. Figure S5. Expanded version of main Figure 6, investigating motion correction stability. (A) De‐trended motion traces and representative navigator images from acquisition with different number of spokes per interleaf. (B) FFT analysis of the detrended motion traces showing a peak at the golden angle frequency. Figure S6. Representative axial and sagittal slices from subject 1, from all motion paradigms, together with the estimated motion traces. The gray region in the time series indicates the short time window at the beginning of the acquisition which is not motion corrected including dummy segments and [file MRM-88-195-s004.pdf]

Motion Corrected Silent ZTE Neuroimaging
Supporting Information

Emil Ljungberg[1,2] | Tobias C. Wood[1] | Ana Beatriz Solana[3] | Steven C.R. Williams[1] | Gareth J. Barker[1] | Florian Wiesinger[1,3]

[1] Department of Neuroimaging, Institute of Psychiatry, Psychology and Neuroscience, King's College London, London, UK

[2] Department of Medical Radiation Physics, Lund University, Lund, Sweden

[3] GE Healthcare, Munich, Germany

SI.1 | Phyllotaxis Trajectory

When designing the 3D phyllotaxis trajectory, the complete spoke distribution is produced by a fixed increment in the azimuthal angle by the golden angle $\phi_G \approx 137.5°$. Subsampling with a factor k produce small angular steps if k is a Fibonacci number (F_n), which is demonstrated in **Figure S1**. The analytical expression valid for $k \in F_n$ is presented in Equation [1] of the main manuscript.

Figure S2 shows Voronoi diagrams of the spoke distribution from the 3D spiral phyllotaxis as formulated by Piccini et al.,[1] and compared to that used in MERLIN, demonstrating a non-isotropic FOV in the Piccini formulation.

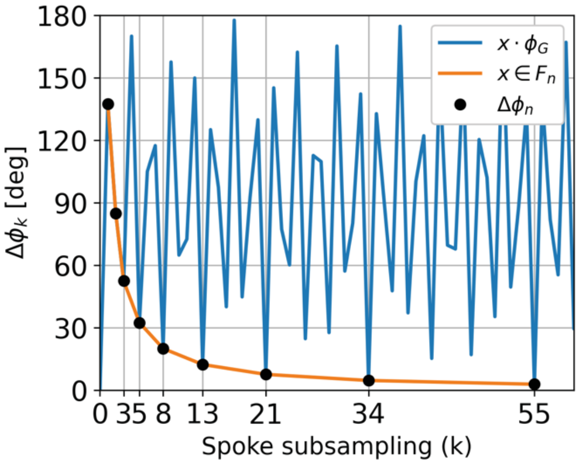

Figure S1 Azimuthal angle increment as a function of spoke subsampling factor. When k is a Fibonacci number, the angular increment ($\Delta\phi_k$) is small, which is required to maintain silent acquisition.

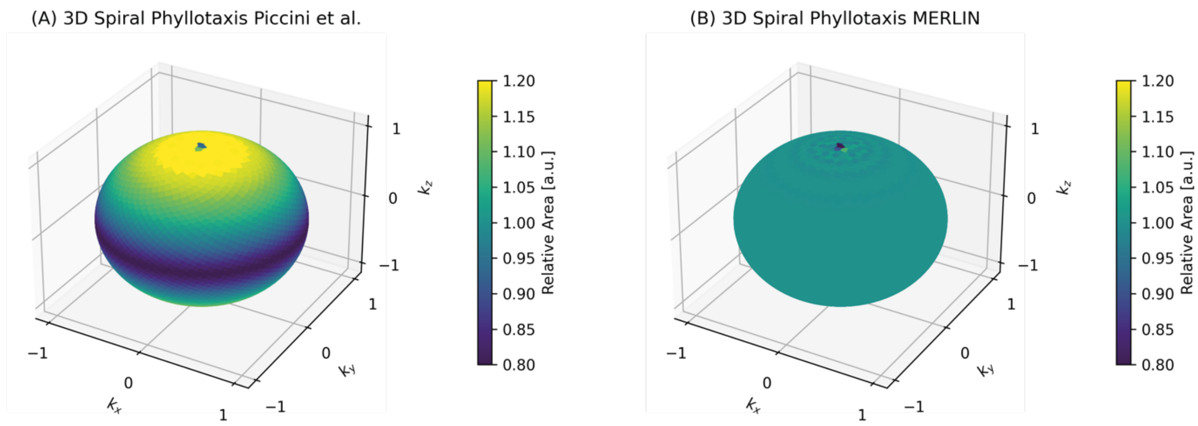

Figure S2 Voronoi diagram which measures the sampling density in k-space. **A** and **B** compares the 3D spiral phyllotaxis trajectory as formulated by Piccini et al. **(A)** with square root modulation of the polar angle θ, resulting in non-uniform sampling density, and the trajectory used in MERLIN **(B)** with a $\cos^{-1}$ modulation, resulting in a linear z-gradient and uniform sampling density for isotropic field of view.

SI.2 | Navigator Resolution

An analysis of the optimal reconstructed resolution for the navigator images is shown in **Figure S3**. Motion corrupted data from one of the subjects in part 3 was processed with navigator resolution from 3-8 mm, followed by TGV reconstruction. The Average Edge Strength (AES) was then calculated for each image and the change relative to the 3 mm experiment was calculated. We observed reduced motion correction quality at lower navigator resolution (**Figure S3A**), which was supported by the quantitative AES analysis in **Figure S3B**.

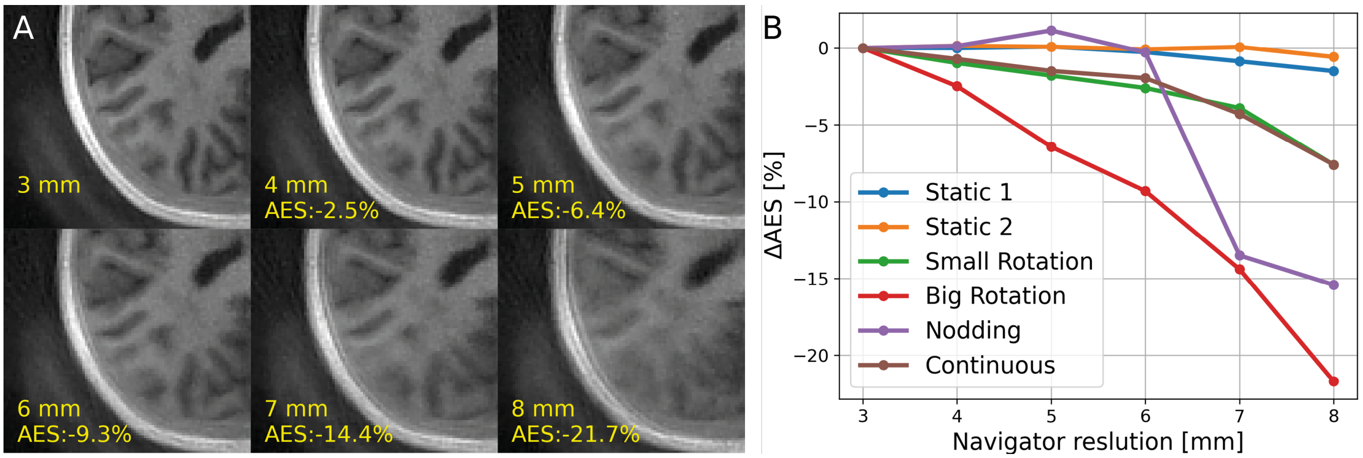

Figure S3 Investigation of motion correction quality as a function of navigator resolution. **(A)** Motion corrected images from the big rotation paradigm, showing reduced motion correction quality with lower navigator resolution. **(B)** Quantitative analysis of image quality using the Average Edge Strength relative to the 3 mm experiment for each motion paradigm. A clear trend to reduced edge strength, i.e., increased blurring, is observed for lower navigator resolution.

SI.3 | Navigator Brain mask

A brain mask was generated using HD-BET[2] to mask out the brain for the registration. The brain mask was twice dilated using FSL maths[3] to include the skull. Example of a brain mask used in one of the experiments is shown in **Figure S4**. White arrows point to visible signal from the headrest and pads which we want to mask out.

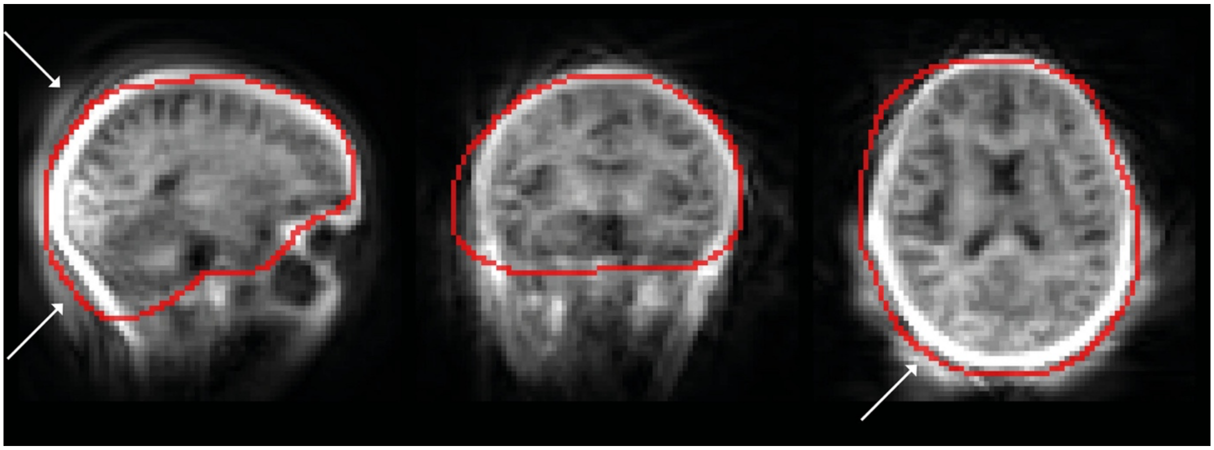

Figure S4 Example of brain mask used for registration. The mask, here shown by outline only, is twice dilated to also cover the skull but to still exclude the signal from the headrest (white arrows) which will confound the registration since it does not move with the head, resulting in a non-rigid motion.

SI.4 | Image Quality Metrics

Average Edge Strength – AES

The average edge strength (AES)[4] is a global measure of the sharpness in the tissue interfaces. A binary edge mask was calculated using a Canny edge filter (with $\sigma = 2$)[5,6] for each axial slice on the first static image for each subject and the edge strength magnitude was calculated using a Sobel filter along all three axes[7]. The AES was then calculated as the mean edge strength within the brain mask for all voxels identified by the Canny edge filter. The AES is sensitive to global intensity scaling, so images were normalised by the 99% percentile image intensity before calculation. The AES is calculated individually for each image, in contrast to the SSIM which is a comparison between two images. We therefore used the percentage difference in AES between the reference and comparison images as our comparison metric

Structural Similarity Index Measurement – SSIM

The structural similarity index measure (SSIM)[8] is a compound measure which compares two images based on intensity, contrast and structure. It is evaluated for each voxel in an image based on a sliding window, from which a mean SSIM (mSSIM) can be calculated. The SSIM was evaluated within the brain mask using the parameters suggested by Wang et al.[8] ($11 \times 11 \times 11$ Gaussian kernel with $\sigma = 1.5$, $K_1 = 0.01, K_2 = 0.03$), to produce an SSIM image, and the mSSIM was then calculated. The advantage of mSSIM is that it is a normalised metric between 0 and 1, and thus directly comparable to other studies. An mSSIM=1 is perfect agreement.

SI.5 | Additional Results

Figure S5 shows an expanded version of Figure 6 from the main manuscript, including the detrended motion traces from each acquisition. **Figure S6-8** shows axial and sagittal slices from all three subjects, with and without motion correction, for all instructed motion paradigms. Images have been bias field corrected and windowed for optimal viewing quality.

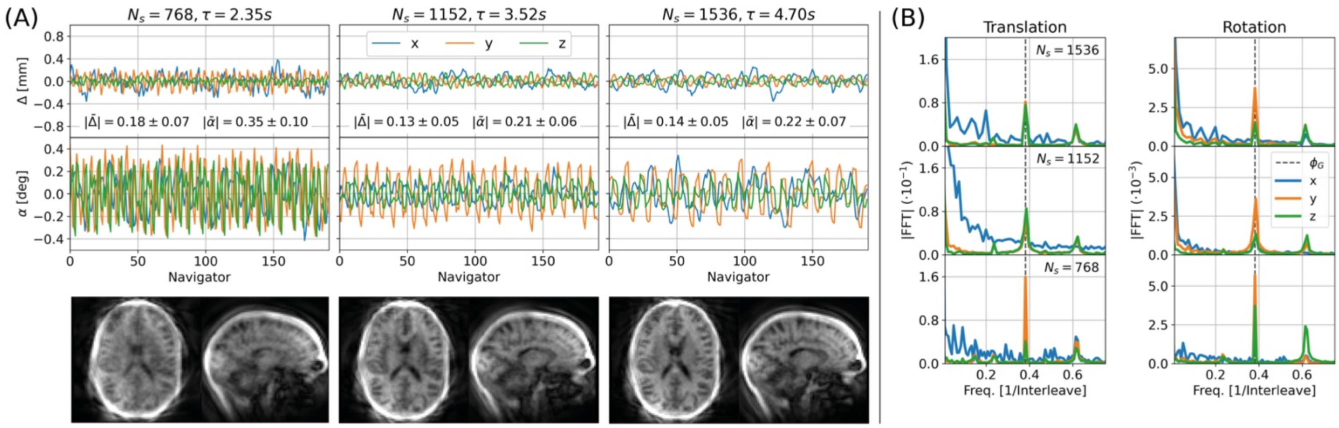

Figure S5 Expanded version of main Figure 6, investigating motion correction stability. **(A)** De-trended motion traces and representative navigator images from acquisition with different number of spokes per interleaf. **(B)** FFT analysis of the detrended motion traces showing a peak at the golden angle frequency.

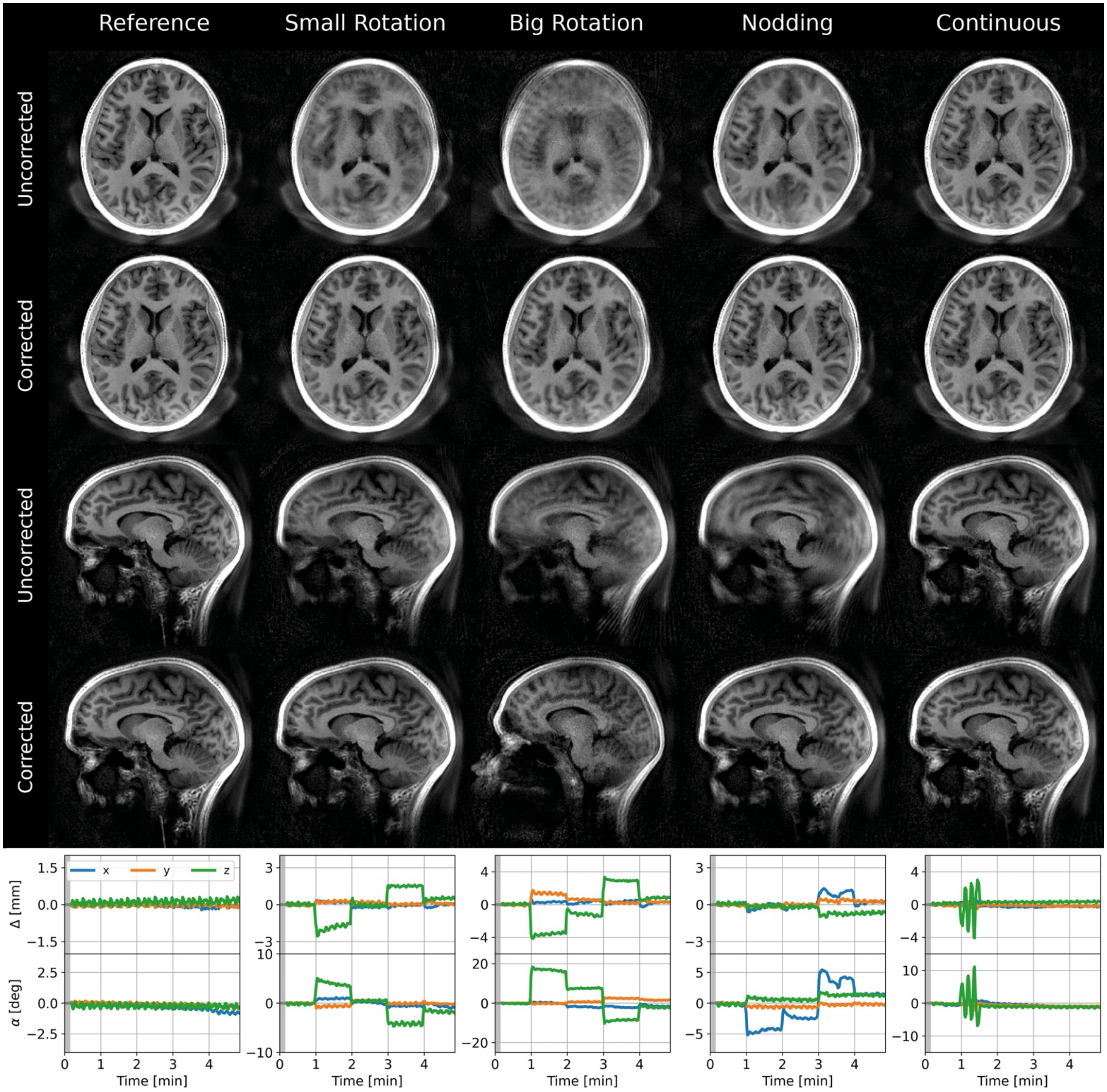

Figure S6 Representative axial and sagittal slices from subject 1, from all motion paradigms, together with the estimated motion traces. The grey region in the time series indicates the short time window at the beginning of the acquisition which is not motion corrected including dummy segments and the low resolution WASPI acquisition.

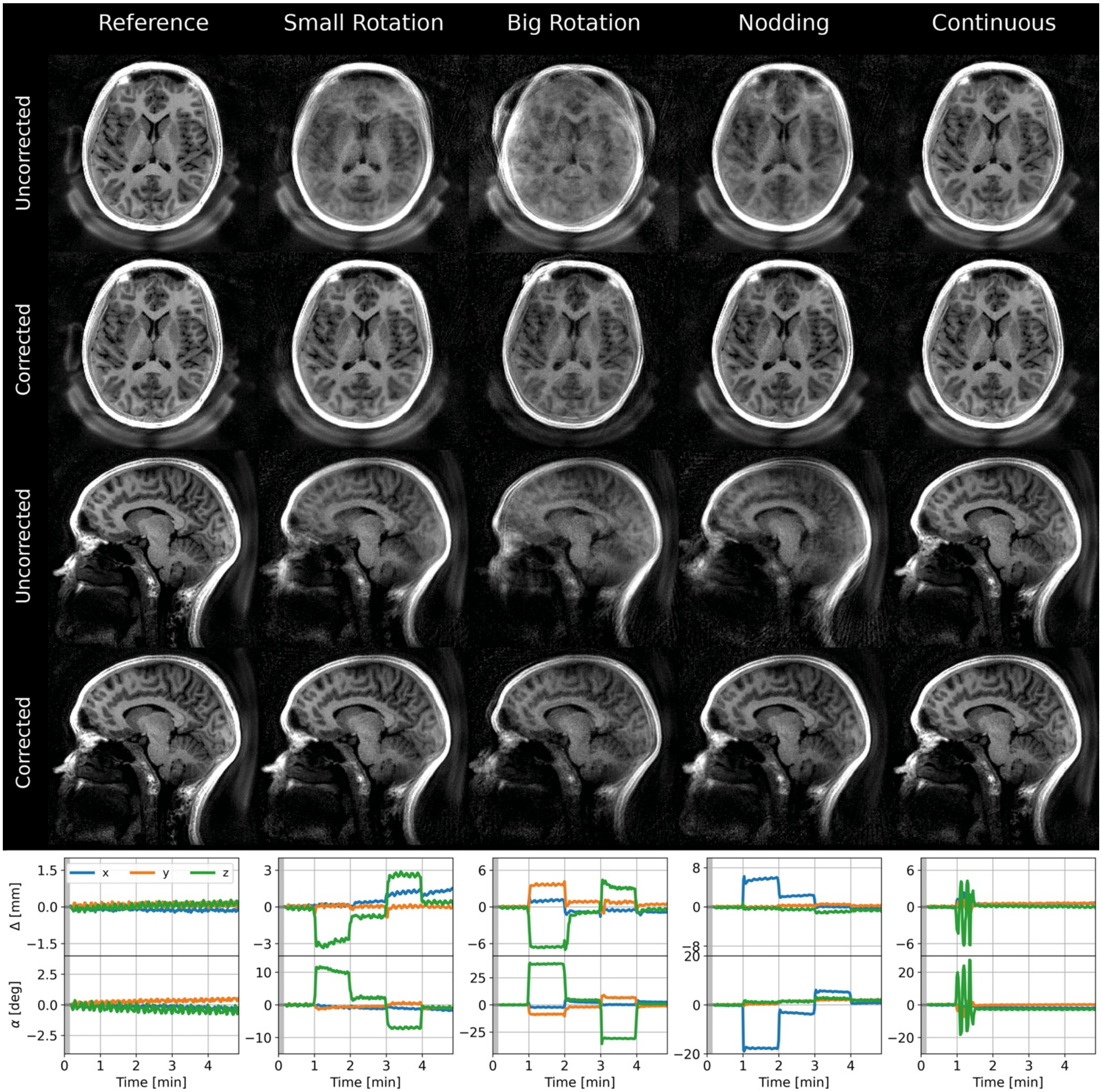

Figure S7 Representative axial and sagittal slices from subject 2, from all motion paradigms, together with the estimated motion traces. The grey region in the time series indicates the short time window at the beginning of the acquisition which is not motion corrected including dummy segments and the low resolution WASPI acquisition.

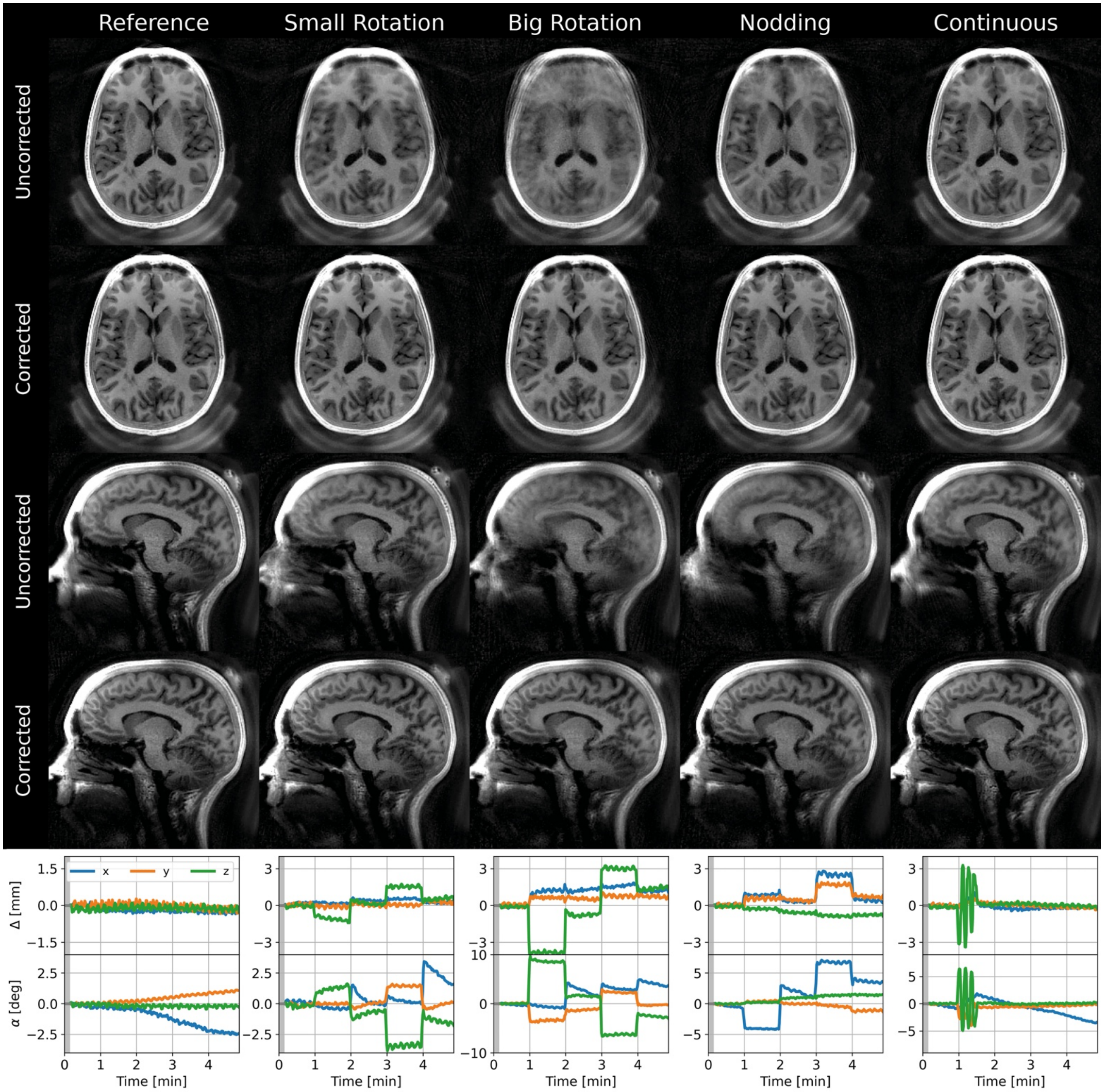

Figure S8 Representative axial and sagittal slices from subject 3, from all motion paradigms, together with the estimated motion traces. The gray region in the time series indicates the short time window at the beginning of the acquisition which is not motion corrected including dummy segments and the low resolution WASPI acquisition.

References

1. Piccini D, Littmann A, Nielles-Vallespin S, Zenge MO. Spiral phyllotaxis: The natural way to construct a 3D radial trajectory in MRI. *Magn Reson Med*. 2011;66(4):1049-1056. doi:10.1002/mrm.22898
2. Isensee F, Schell M, Pflueger I, et al. Automated brain extraction of multisequence MRI using artificial neural networks. *Hum Brain Mapp*. 2019;40(17):4952-4964. doi:10.1002/hbm.24750
3. Jenkinson M, Beckmann CF, Behrens TEJ, Woolrich MW, Smith SM. Fsl. *Neuroimage*. 2012;62(2):782-790. doi:10.1016/j.neuroimage.2011.09.015
4. Aksoy M, Forman C, Straka M, Çukur T, Hornegger J, Bammer R. Hybrid prospective and retrospective head motion correction to mitigate cross-calibration errors. *Magn Reson Med*. 2012;67(5):1237-1251. doi:10.1002/mrm.23101
5. Van Der Walt S, Schönberger JL, Nunez-Iglesias J, et al. Scikit-image: Image processing in python. *PeerJ*. 2014;2014(1):1-18. doi:10.7717/peerj.453
6. Canny J. A Computational Approach to Edge Detection. *IEEE Trans Pattern Anal Mach Intell*. 1986;PAMI-8(6):679-698. doi:10.1109/TPAMI.1986.4767851
7. Kroon D-J. Numerical optimization of kernel based image derivatives. *Short Pap Univ Twente*. 2009. http://www.k-zone.nl/Kroon_DerivativePaper.pdf.
8. Wang Z, Bovik AC, Sheikh HR, Simoncelli EP. Image quality assessment: From error visibility to structural similarity. *IEEE Trans Image Process*. 2004;13(4):600-612. doi:10.1109/TIP.2003.819861
